# Fish Community Structure, Diversity, and Their Environmental Correlates in Two Arid-Region Lakes of Northwest China: Bosten Lake and Taitema Lake

**DOI:** 10.3390/ani16193149

**Published:** 2026-10-08

**Authors:** Yuqing Zhang, Jingru Yang, Wenrui Jiang, Liang Wu, Hongyu Ma, Jing Song, Weimin Wang, Jie Wei, Zhulan Nie

**Affiliations:** 1 College of Life Science and Technology, Tarim University, Alar 843300, China; 2 State Key Laboratory Breeding Base of Protection and Utilization of Biological Resources in Tarim Basin, Xinjiang Production and Construction Crops and Ministry of Science and Technology, Alar 843300, China; 3 Tarim Rare Fish Research Center, Aral 843300, China; 4 College of Fisheries, Huazhong Agricultural University, Wuhan 430070, China

**Keywords:** fish diversity, arid lakes, environmental factors, Bosten Lake, Taitema Lake

## Abstract

Bosten Lake and Taitema Lake are important inland lakes in arid Northwest China, but recent fish-assemblage variation in these systems remains poorly resolved. We surveyed fish communities at 10 sites in Bosten Lake and four sites in Taitema Lake from 2023 to 2025 using standardized conventional capture methods. Across the three years, 20 species from 7 orders and 12 families were recorded in Bosten Lake and 12 species from 6 orders and 9 families in Taitema Lake; Cyprinidae was the most species-rich family in both lakes. The cumulative observed species pool and effort-standardized richness were higher in Bosten Lake, but year-specific site-level richness contrasts were not significant after Holm adjustment. Community composition differed between lakes after adjustment for year using exact whole-site permutations (Bray–Curtis PERMANOVA: pseudo-F1,38 = 37.92, partial R^2^ = 0.499, *p* = 0.0010), although multivariate dispersion also differed (PERMDISP: F = 5.46, *p* = 0.030). Site-restricted PERMANOVA detected year effects in Bosten Lake (pseudo-F2,18 = 29.37, partial R^2^ = 0.765, *p* < 0.001) and Taitema Lake (pseudo-F2,6 = 156.36, partial R^2^ = 0.981, exact *p* = 0.0046). Bosten Lake had higher Margalef richness (Taitema minus Bosten: −0.818, 95% CI −1.451 to −0.185, *p* = 0.016) and Pielou evenness (−0.056, 95% CI −0.105 to −0.008, *p* = 0.027), whereas Shannon–Wiener diversity did not differ detectably between lakes (−0.169, 95% CI −0.673 to 0.335, *p* = 0.478). After controlling for site and year, no diversity–environment correlation remained significant after false-discovery-rate correction, and the partial RDA test of within-site temporal environmental–community covariation was nonsignificant (pseudo-F2,24 = 1.285, partial R^2^ = 0.097, *p* = 0.1754). Thus, the study documents clear spatial separation between lakes and interannual variation within lakes, but does not identify the retained environmental variables as statistically robust drivers of those patterns.

## Simple Summary

Bosten Lake and Taitema Lake are two important lakes in arid Northwest China. From 2023 to 2025, we surveyed fish assemblages and measured environmental conditions. We recorded 20 species in Bosten Lake and 12 in Taitema Lake, with Cyprinidae being the most species-rich family in both lakes. Assemblage composition differed between lakes and changed significantly among years within each lake, indicating pronounced temporal species replacement. Bosten Lake had higher Margalef richness and Pielou evenness overall, whereas Shannon–Wiener diversity did not differ significantly between lakes. After accounting for repeated sites and years and correcting for multiple testing, no robust association was detected between the retained environmental variables and either fish-diversity indices or assemblage composition. These results document contrasting fish assemblages and rapid interannual turnover, while showing that longer verified hydrological and environmental series are needed to identify causal drivers.

## 1. Introduction

Fish, as a key functional group in aquatic ecosystems, occupy multiple trophic levels in the food web and exert profound effects on community structure and the aquatic environment through trophic cascade effects [1]. However, freshwater fish are facing an unprecedented crisis worldwide. It is estimated that nearly one-third of all freshwater fish species are at risk of extinction [2]. The ongoing decline in fish resources not only threatens the stability and sustainable development of aquatic ecosystems, but also poses a serious challenge to regional biodiversity conservation. Therefore, conducting comprehensive and accurate monitoring and assessment of fish diversity has become an essential prerequisite for the conservation of fish resources and the maintenance of ecosystem health.

For a long time, fish diversity assessment has mainly depended on conventional capture methods, including gillnetting, electrofishing, and visual identification. Systematic morphological surveys are essential for gathering baseline community data, enabling long-term monitoring and comparative studies, and supplying fundamental information for the conservation and management of fish resources. In addition, these traditional approaches provide direct biological data (e.g., body length and population structure) that eDNA methods cannot offer. Hence, conventional capture and morphological surveys remain valuable complements to eDNA-based monitoring in fish diversity monitoring [3]. Arid-region lakes are situated in extreme environments characterized by scarce precipitation and intense evaporation, rendering their ecosystems highly fragile. As an important means of obtaining baseline data on fish communities and understanding species composition and population structure, traditional morphological surveys have been widely applied in studies of arid-region lakes both in China and internationally. Changes in fish communities over the past 30 years in the Wadi El-Rayan arid lakes of Egypt were analyzed by Abdelhady, et al. [4] based on catch composition data, and it was found that lake shrinkage was accompanied by a shift in catch composition from pelagic-dominant to demersal-dominant species, revealing multiple impacts driven by climate warming and human mismanagement, including alterations in fish community structure, interannual shifts in dominant species, and overfishing risks to target fish. Saç, et al. [5] conducted a study in the Burdur endorheic basin of Turkey using traditional methods such as gillnetting and electrofishing, capturing 15 fish species, of which nearly half were non-native, indicating the profound effects of water scarcity and habitat fragmentation on fish communities. Farrag and AbdAllah [6] collected 26 specimens of Nile perch (*Lates niloticus*) from Lake Nasser and the Nile River, respectively, and through traditional morphometric and meristic analyses, demonstrated that environmental differences influenced fish morphology. Zhang, et al. [7] collected samples of *Gymnodiptychus dybowskii* from the Manas River and the Tekes River in 2020–2021, identified their morphological characteristics using fish ecological methods, and analyzed morphological differences among different geographical populations, revealing pronounced differentiation in external features and significant differences in body weight and body length (*p* < 0.05) between the two geographic populations. The above studies demonstrate that traditional morphological survey methods provide important information for assessing fish-community responses and revealing the responses of fish communities to environmental changes in arid-region lakes. However, systematic and continuous surveys on fish community structure and diversity in arid-region lakes remain relatively limited, and the patterns of community turnover and their key environmental correlates are still poorly understood, underscoring the urgent need for further in-depth research.

Bosten Lake is the largest inland freshwater lake in Xinjiang and one of the most representative lakes in arid and semi-arid regions. Its primary water source is the Kaidu River, and the lake exhibits a salinity gradient decreasing from northwest to southeast. The fish community structure and historical compositional changes in Bosten Lake are of significant research value in the context of water-level fluctuations, salinity gradients, and the introduction of non-native species. Taitema Lake, as the terminal lake of the Tarim River, serves as a critical node for ecological water conveyance in the Tarim River Basin. Its ecological restoration holds important indicative significance for the health of the lower reaches of the Tarim River [8], and it plays an irreplaceable role in preventing the convergence of the Taklimakan and Kumtag deserts, mitigating land desertification, and protecting regional biodiversity [9]. However, under the dual pressures of climate change and human activities, the fish resources of Bosten Lake have been in constant flux since the onset of commercial fishing in 1958 and the introduction of non-native fishes in 1962, with native species gradually being replaced by exotic species. In Taitema Lake, river flow cessation led to the functional extinction of endemic fish species in 1980. Since the implementation of ecological water conveyance in 2001, the lake area has gradually recovered [10], and the fish community is now dominated by artificially stocked and naturally dispersed populations, though habitat stability remains relatively weak [11,12]. Although studies on fish resources and diversity in both lakes have been reported, most have been based on single-year or short-term surveys [13], lacking systematic analyses using multi-year continuous data; consequently, the patterns of community turnover and their key environmental correlates remain unclear. Therefore, based on survey data from 2023 to 2025, this study aims to analyze the spatiotemporal variations in fish community structure and diversity in the two lakes, characterize their interannual compositional dynamics and their associations with environmental factors, and provide an observational basis for fish resource conservation and aquatic ecological restoration in arid-region lakes.

## 2. Materials and Methods

### 2.1. Study Area and Sampling Site Layout

Bosten Lake is located in the hinterland of Xinjiang, with geographical coordinates ranging from 41°49′ to 42°09′ N and 86°42′ to 87°26′ E, and a water area of approximately 1210.5 km^2^. It is an important fishery base and an ecological security barrier in western China [14]. Taitema Lake is situated on the southeastern margin of the Tarim Basin, with geographical coordinates ranging from 39°05′ to 39°45′ N and 87°20′ to 88°40′ E, and a water area of approximately 88 km^2^. As a terminal lake receiving water from the Tarim and Qarqan Rivers, its water area has varied substantially in response to river inflow and ecological water conveyance [15].

**Figure 1:**
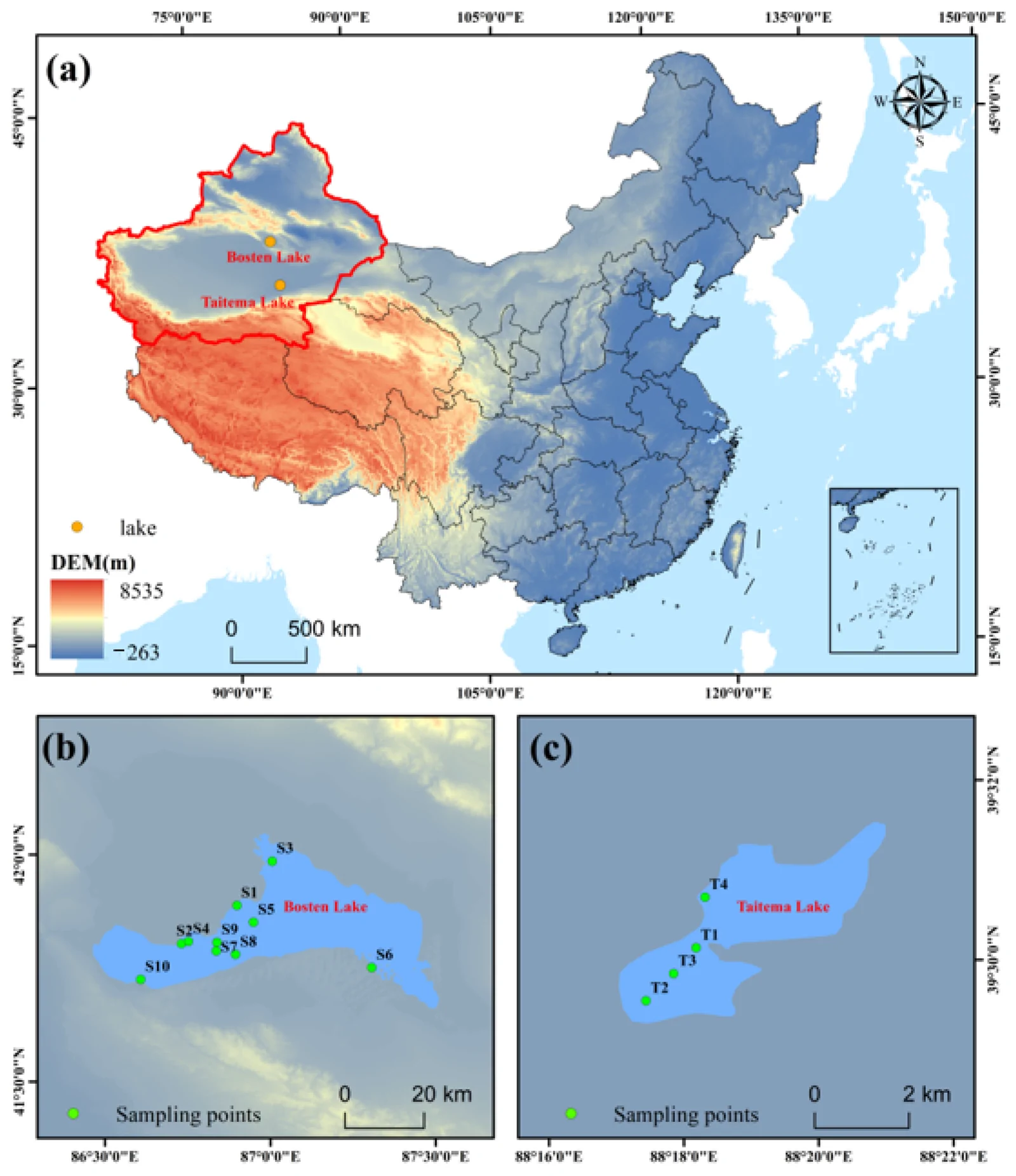
Location of the study lakes and sampling sites. (**a**) Geographical overview of Bosten Lake and Taitema Lake in northwest China, with a digital elevation model (DEM) as the base map. (**b**) Spatial distribution of sampling sites in Bosten Lake. (**c**) Spatial distribution of sampling sites in Taitema Lake.

Based on a comprehensive consideration of lake topography, water depth, shoreline characteristics, and the spatial distribution of inlets and outlets, combined with field reconnaissance, a total of 10 sampling sites (S1–S10) were established in Bosten Lake, covering the lake center, nearshore areas, river inlets, and drainage outlets. In Taitema Lake, 4 sampling sites (T1–T4) were set up to cover the main water areas of the lake. From 2023 to 2025, fish sampling was conducted once annually during the available wet-season survey window (June–August). Dry-season sampling was not attempted because water-level recession exposes extensive mudflats, disconnects fixed sampling sites, and prevents consistent deployment of the standardized gear protocol across all sites. The geographical distribution of all sampling sites is illustrated in Figure 1.

### 2.2. Fish Sample Collection, Identification, and Environmental Factor Measurement

Three types of fishing gear were deployed at each site: 1. Three-layer gillnets: each set measured 30 m in length × 1.5 m in height, with three mesh sizes (1.5, 3, 5 cm) to cover different fish size classes. Gillnets were set near the bottom and retrieved after 12 h of deployment. 2. Cage traps: each trap was 0.5 × 0.5 × 1 m with 1 cm mesh size. Three replicate traps were deployed per site and soaked for 12 h, matching the gillnet deployment period. 3. Seine net: 10 m in length × 1.5 m in height. One single 30-m shore-parallel haul was performed at each site as instantaneous sampling, with no long-term soaking. The estimated swept area was approximately 45 m^2^ per haul. Individual counts cannot be partitioned by sampling gear, as gear-related metadata were not documented during field sampling. An identical multi-gear sampling protocol was applied consistently across all sites and years to ensure comparable sampling effort.

All field sampling and fish collection were conducted with the approval of the local fishery administration and lake management authorities, in compliance with regional fishery management regulations. Captured fish were immediately transferred to aerated water containers for temporary holding on site. All field sampling and fish collection were conducted with the approval of the local fishery administration and lake management authorities, in compliance with regional fishery management regulations. Captured fish were immediately transferred to aerated water containers for temporary holding on site. All individuals were first anaesthetised by immersion in tricaine methanesulfonate (MS-222, 35 mg/L, Macklin Biochemical Co., Ltd., Shanghai, China); this induction concentration was specified in the approved institutional protocol. Selection of MS-222 (Macklin Biochemical Co., Ltd., Shanghai, China) dose and exposure time is species-, size-, temperature-, and context-dependent [16]. Following loss of opercular movement and reflex response, which confirmed deep anaesthesia, morphological identification, total length, standard length, and body weight measurements were performed on fresh specimens. After measurement, euthanasia was completed by continued immersion in the same MS-222 solution, with fish maintained in the solution for at least 10 min after cessation of all opercular movement, consistent with the minimum post-apnoea exposure endpoint specified in the AVMA Guidelines for the Euthanasia of Animals [17]. No live fish were directly placed into fixative. Specimens that could be reliably identified to species level in the field were retained as voucher specimens. Specimens that were unidentifiable or taxonomically ambiguous in the field were preserved in 10% neutral buffered formalin or 95% ethanol for subsequent laboratory taxonomic confirmation. All fish-handling procedures complied with the institutional animal ethics approval (Approval No. PB20230527001) and followed standard humane fish research protocols. Taxonomic identification was performed using regional taxonomic references, including Fishes of Xinjiang [16]. Native and introduced fish species were distinguished, and the taxonomic rank of each species was clearly determined. No live fish were directly placed into fixative. Specimens that could be reliably identified to species level in the field were retained as voucher specimens. Specimens that were unidentifiable or taxonomically ambiguous in the field were preserved in 10% neutral buffered formalin or 95% ethanol for subsequent laboratory taxonomic confirmation. All fish-handling procedures complied with the institutional animal ethics approval (Approval No. PB20230527001) and followed standard humane fish research protocols. Taxonomic identification was performed using regional taxonomic references, including *Fishes of Xinjiang* [18]. Native and introduced fish species were distinguished, and the taxonomic rank of each species was clearly determined. Water-quality measurements were obtained synchronously with fish sampling at each site-year. Water temperature, pH, dissolved oxygen, and salinity were measured in situ at 0.5 m depth with a calibrated YSI Professional Plus multiparameter sonde (Xylem Analytics, Beijing, China), and Secchi depth was measured with a standard Secchi disk. Samples for laboratory analyses were collected without filtration in separate polyethylene bottles. Aliquots for total alkalinity were kept at 4 ± 2 °C and analysed within 14 d, whereas aliquots for total nitrogen were frozen at −20 °C and analysed within one month. Total alkalinity was determined by automatic potentiometric acid titration with a ZDJ-4A automatic potentiometric titrator (INESA Scientific Instrument Co., Ltd., Shanghai, China), following SL 83—1994. A 100 mL aliquot was titrated with standardised 0.0250 mol L^−1^ HCl to pH 4.4–4.5, and the result was calculated as mg L^−1^ CaCO_3_. Because SL 83—1994 does not specify a universal method detection limit, an operational reporting limit of 2 mg L^−1^ as CaCO_3_ was used. Total nitrogen was determined using the alkaline potassium persulfate digestion UV spectrophotometric method (HJ 636—2012). A 10.00 mL aliquot was mixed with 5.00 mL alkaline potassium persulfate, digested at 120–124 °C for 30 min, acidified with 1.0 mL HCl (1 + 9), diluted to 25 mL, and measured at 220 and 275 nm using a UV–visible spectrophotometer (Beijing Purkinje General Instrument Co., Ltd., Beijing, China) with a 10 mm quartz cell. The corrected absorbance was A220−2A275; the method detection limit was 0.05 mg L^−1^ as N and the calibrated working range was 0.20–7.00 mg L^−1^. Total alkalinity was reported as mg L^−1^ as CaCO_3_ and total nitrogen as mg L^−1^. Field blanks and duplicate samples were included for quality assurance. Environmental observations were paired with fish data at the site-year level. Variance inflation factors were used to retain predictors with VIF < 10. For the diversity–environment correlations, *p* values were adjusted within each lake across the 21 tests (seven retained environmental variables × three diversity indices) using the Benjamini–Hochberg false-discovery-rate procedure. The corresponding measurement indicators and units are listed in Supplementary Table S2.

### 2.3. Calculation of Community Diversity Indices

#### 2.3.1. Species Relative Abundance

The annual relative abundance of each species was calculated based on the annual catch count of individuals per species:

*RA_i_*_,*y*_ = *n_i_*_,*y*_/*N_y_*

where *RA_i_*_,*y*_ is the relative abundance of species *_i_* in year *_y_*; *n_i_*_,*y*_ is the total number of individuals of species *_i_* captured in year *_y_*; and *N_y_* is the total number of fish individuals captured from the lake in year *_y_*.

#### 2.3.2. Community Diversity Indices

Diversity indices were calculated at both the lake-year level (for descriptive trend analysis) and the site-year level (for statistical inference to preserve the replicated sampling design). Three classical community evaluation indices were selected to characterize the fish community structure:

1. Shannon–Wiener diversity index (*H′*): *H′* = −Ʃ*P_i_*ln*P_i_*
2. Margalef richness index (*D*): *D* = (*S* − 1)/ln*N*
3. Pielou evenness index (*J*): *J* = *H′/H*_max_, *H*_max_ = ln*S*

where *S* is the total number of fish species collected in the lake in a given year; *N* is the total number of individuals collected in that year (for site-level calculation, *S* is the number of species and *N* is the total number of individuals captured at a given sampling site in a given year); *P_i_* is the proportion of individuals of species *_i_* to the total number of all fish individuals; and *H_max_* is the maximum possible diversity index of the community.

#### 2.3.3. Dominant Species Determination

For this study, species with an annual individual proportion *Di* ≥ 0.10 were classified as annual dominant species:

*D_i_* = *n_i_*/*N*

where *D_i_* is the dominance index of species *_i_*; *n_i_* is the annual number of individuals of species *_i_* captured; and *N* is the total number of individuals captured in that year.

### 2.4. Data Analysis

Statistical analyses were performed in R 4.3.0 and SPSS 26.0 (α = 0.05 significance level). Two complementary standardization methods were implemented to handle unequal sampling effort across lakes. Individual-based rarefaction was computed on per-lake pooled abundance data using the exact hypergeometric method to estimate expected species richness under sampling-without-replacement for given individual counts. Sample-based species-accumulation curves were produced with 1000 random permutations of sampling-site order, yielding mean cumulative richness and 95% empirical intervals per site number. Linear mixed-effects models were fitted separately to the site-year Shannon–Wiener, Pielou, and Margalef indices. Lake and categorical year were fixed effects, and site nested within lake was a random intercept. REML was used for coefficient and variance-component estimation. Lake contrasts (Taitema minus Bosten) were tested with 12 between-site denominator degrees of freedom and reported with 95% confidence intervals. Likelihood-ratio sensitivity tests compared models refitted by ML. Residual Q–Q and residual-versus-fitted plots, Shapiro–Wilk and Breusch–Pagan tests, standardized residuals, and leave-one-site-out refits were used for model diagnostics; lake-specific residual variances were examined in a sensitivity analysis. Community composition was analysed from square-root-transformed relative abundances using Bray–Curtis dissimilarities. A two-dimensional NMDS was used for visualization. For the between-lake PERMANOVA, the formula was Bray–Curtis distance ~ Year + Lake, and the marginal lake term was tested after adjustment for year. Lake labels were permuted at the site (whole-plot) level: all three observations from a site retained the same permuted lake label. All 1001 assignments of four of the 14 sites to Taitema Lake, preserving the observed 10:4 site allocation, were evaluated. For temporal tests within each lake, the formula was Bray–Curtis distance ~ Site + Year, with year tested after adjustment for site. Year labels were permuted independently within each site, keeping observations within their original site. The Bosten Lake test used 9999 restricted random permutations; the Taitema Lake test used all 6^4^ = 1296 possible within-site permutations.

Fish-community diversity indices (Shannon–Wiener, Margalef richness, Pielou evenness) were calculated for each site-year to match the observational scale of environmental data. Repeated-measures partial Pearson correlations were calculated separately for each lake at the site-year level (Bosten Lake: *n* = 30; Taitema Lake: *n* = 12), controlling for site and year. After VIF screening (VIF < 10), seven environmental variables were retained: Secchi depth, water temperature, pH, dissolved oxygen, salinity, total alkalinity, and total nitrogen. Within each lake, the 21 *p* values from seven environmental variables × three diversity indices were adjusted using the Benjamini–Hochberg false-discovery-rate procedure. For the descriptive environmental PCA, the seven retained variables were not transformed; each variable was centred and scaled once across all 42 site-year observations (global z-score standardisation), thereby retaining between-lake mean differences. The first two PCA scores were used as environmental predictors in a partial RDA of the Hellinger-transformed species-abundance matrix: Hellinger community ~ PC1 + PC2 + Condition (Site + Year). Conditioning on site removed all persistent between-site variation, including the lake-level contrast because every site belonged to one lake; conditioning on year removed variation shared by sites in a given year. The remaining hypothesis was therefore whether within-site temporal departures in community composition covaried with within-site temporal departures along the environmental PCA gradients, after removal of common year effects. Significance was assessed with 9999 permutations of the conditioned community residuals restricted within site. Lake-level annual pooled diversity values are used only for descriptive purposes and were not included in any significance testing. For the exploratory species–environment analysis, site-year species relative abundances were Hellinger transformed, and both each species vector and each of the seven retained environmental variables were residualised against site and categorical year across all 42 site-year observations. Partial Pearson coefficients were then calculated from the residuals. Only species occurring in at least eight site-year samples and represented by at least 20 individuals were included. *p* values were obtained from 9999 permutations restricted within sites, and the 77 *p* values (11 species × seven variables) were adjusted together using the Benjamini–Hochberg procedure. Species-composition plots, relative-abundance stacked bars, diversity-index line plots, and correlation heatmaps were visualized in Origin 2021.

## 3. Results

### 3.1. Species Composition Characteristics of Fish Communities in the Two Lakes

**Figure 2:**
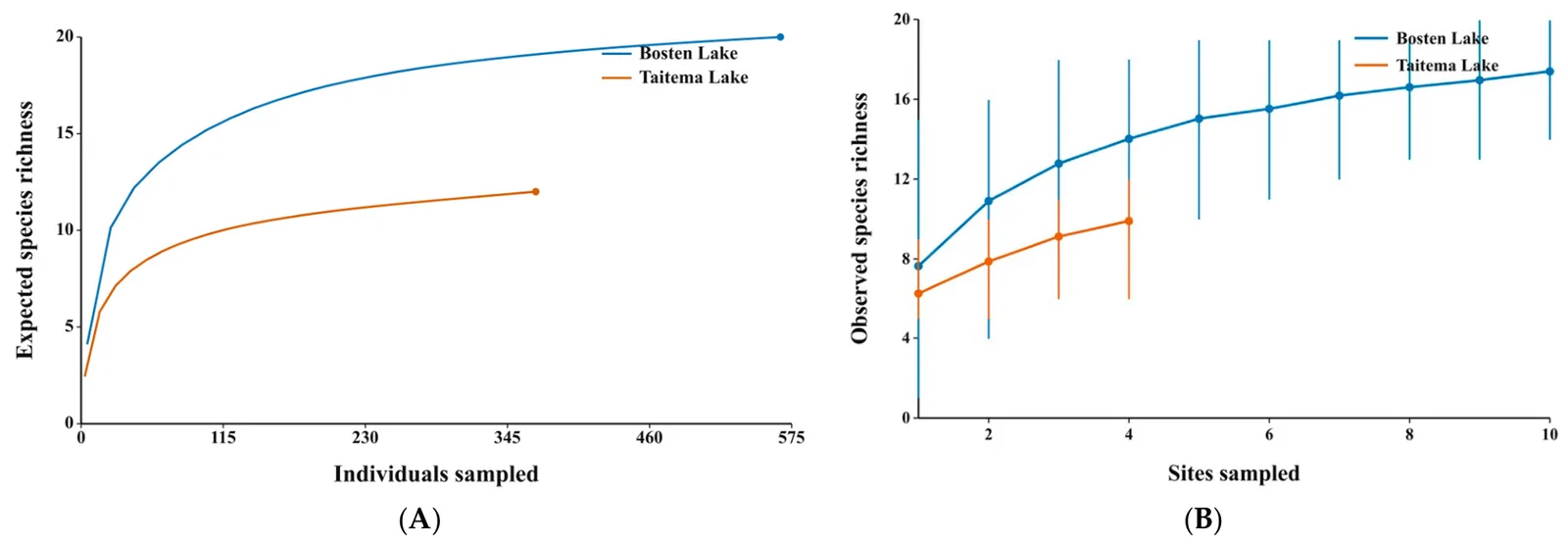
Sampling-effort standardization of fish species richness in Bosten Lake and Taitema Lake, 2023–2025. (**A**) Individual-based rarefaction curves showing expected richness against individuals sampled. (**B**) Sample-based accumulation curves showing cumulative observed richness against sites sampled; error bars are 95% empirical intervals from 1000 randomized site orders.

Individual-based rarefaction curves increased rapidly at low sampling effort and gradually neared an asymptote for both lakes (Figure 2). Within the shared range of sampled individuals, Bosten Lake exhibited higher expected species richness than Taitema Lake. Sample-based accumulation curves increased with sampling-site count: 20 species with a total of 564 individuals were observed for Bosten Lake (10 sites) versus 12 species with a total of 368 individuals for Taitema Lake (4 sites). The richness disparity is therefore not purely driven by Bosten Lake’s greater number of sites.

**Figure 3:**
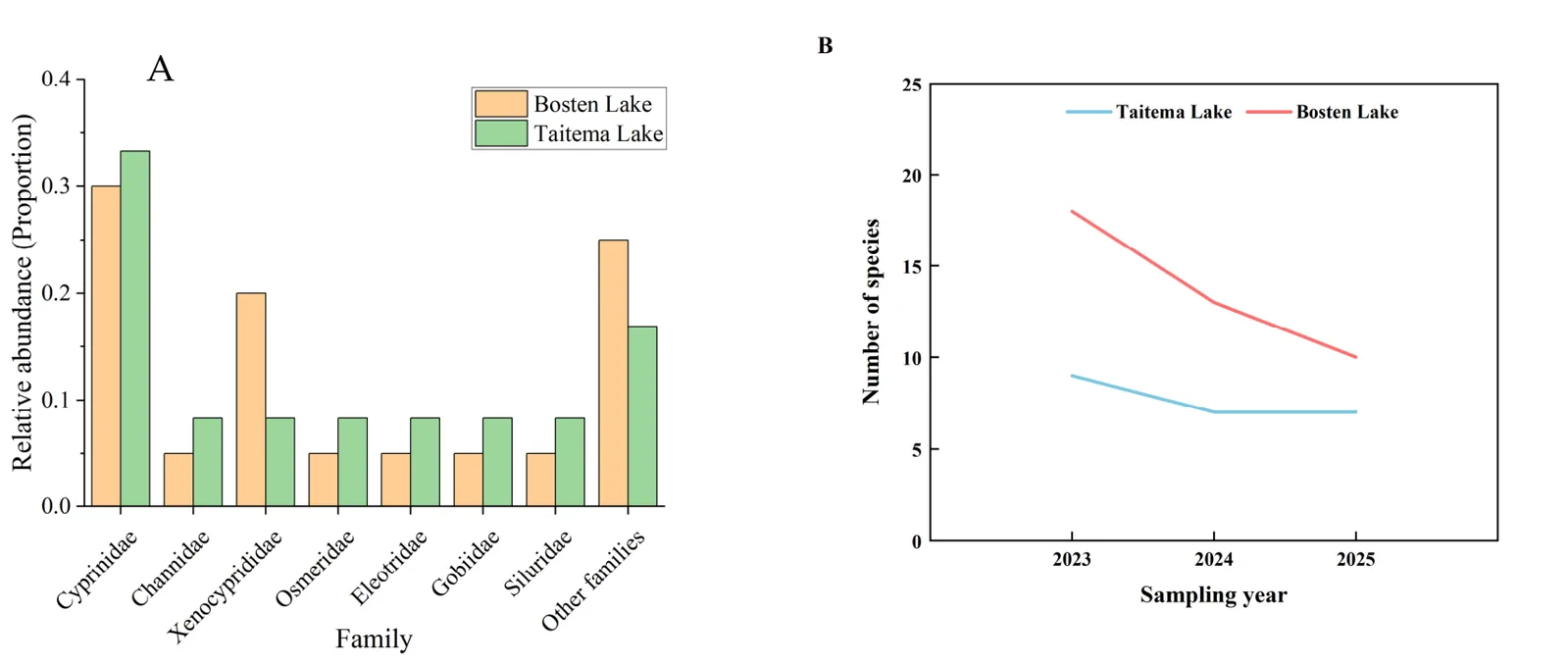
(**A**) Family-level composition of fish species in Bosten Lake and Taitema Lake over three consecutive years (2023–2025). (**B**) Species-level richness of fish species in Bosten Lake and Taitema Lake over three consecutive years (2023–2025).

Across 2023–2025, the cumulative observed species pool comprised 20 species from 12 families in Bosten Lake and 12 species from 9 families in Taitema Lake. Cyprinidae was the most species-rich family in both lakes, accounting for 30.0% and 33.3% of cumulative recorded species, respectively (Figure 3).

**Figure 4:**
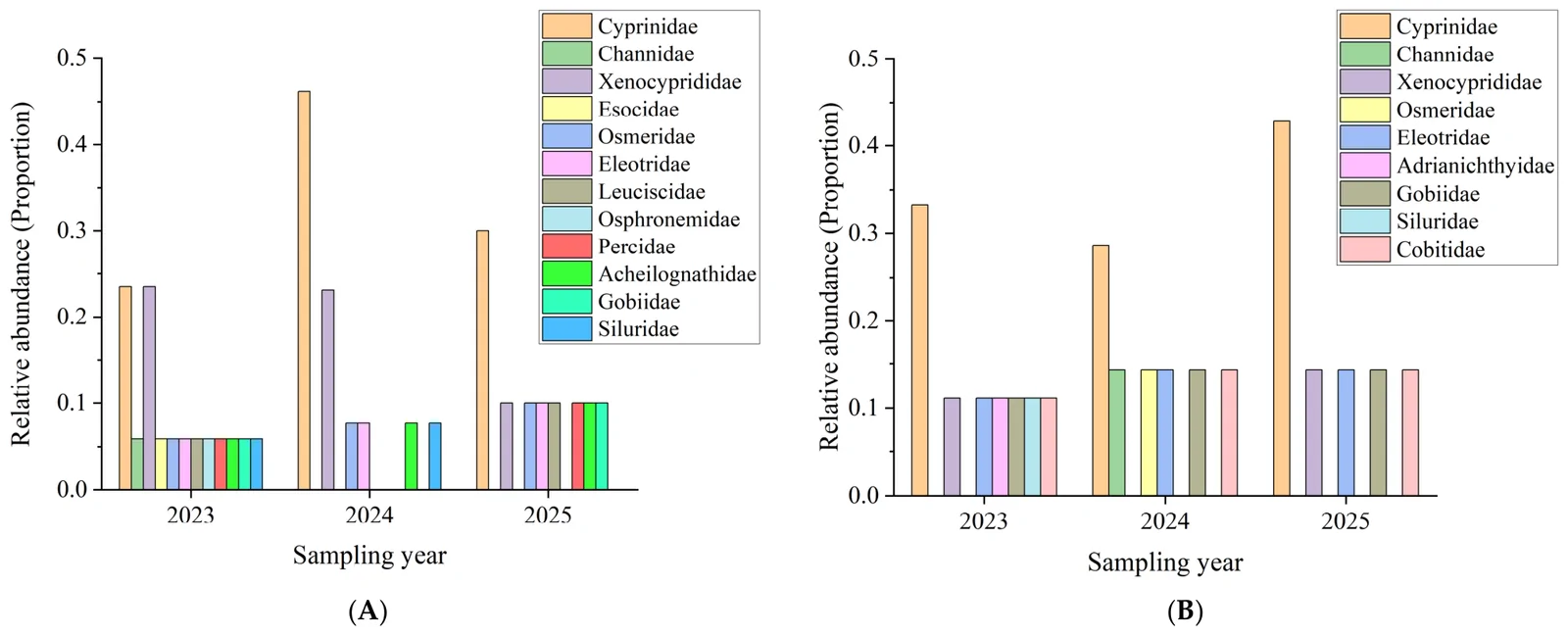
Family-level composition of fish communities in Bosten Lake (**A**) and Taitema Lake (**B**) from 2023 to 2025.

Species richness in both lakes showed a decreasing trend over the study period, with the highest number of species recorded in 2023 (18 species in Bosten Lake and 9 species in Taitema Lake). In 2023, a total of 18 fish species were recorded in Bosten Lake (Figure 4A), belonging to 7 orders and 12 families. Among them, Cyprinidae and Xenocyprididae each comprised 4 species, accounting for 22.2% of the total number of species, while each of the remaining families contained only 1 species, contributing 5.5% individually. In 2024, a total of 13 fish species were recorded, belonging to 4 orders and 6 families, of which Cyprinidae accounted for 6 species (46.1%) and Xenocyprididae for 3 species (23.0%). In 2025, a total of 10 fish species were recorded, belonging to 4 orders and 8 families, with Cyprinidae comprising 3 species (30.0%) and each of the other families contributing 1 species (10.0%). Across the three years, Cyprinidae remained the most species-rich family, with its proportional representation consistently exceeding 22.0%.

In 2023, a total of 9 fish species were recorded in Taitema Lake (Figure 4B), belonging to 4 orders and 7 families, of which Cyprinidae comprised 3 species, accounting for 33.3% of the total number of species, while each of the remaining families contained only 1 species, contributing 11.1% individually. In 2024, a total of 7 fish species were recorded, belonging to 4 orders and 6 families, with Cyprinidae accounting for 2 species (28.5%), and each of the other families contributing 1 species (14.2%). In 2025, a total of 7 fish species were recorded, belonging to 2 orders and 5 families, of which Cyprinidae comprised 3 species (42.8%), with each of the remaining families contributing 1 species (14.2%). Across the three years, the proportion of Cyprinidae remained consistently above 28.0%, establishing it as the most species-rich family.

Annual changes in species relative abundance were described without assigning salinity–alkalinity tolerance or sensitivity categories. In Bosten Lake, *Pseudorasbora parva* was most abundant in 2023, whereas *Hypomesus olidus* became most abundant in 2024 and remained abundant in 2025. In Taitema Lake, *Hemiculter leucisculus* was most abundant in 2023, whereas *Triplophysa yarkandensis* was most abundant in 2024 and 2025. Exploratory partial correlations between the 11 common species and all seven retained environmental variables were evaluated (Figure S2), but none of the 77 relationships remained significant after false-discovery-rate correction (all q > 0.05). Therefore, these coefficients provide no statistical basis for classifying any taxon as tolerant, moderately tolerant, or sensitive, and no such classification is made in this study.

### 3.2. Fish Relative Abundance and Interannual Turnover of Dominant Species

Across the full 2023–2025 catch, *Hypomesus olidus* (16.8%) and *Carassius auratus* (12.9%) had the highest cumulative relative abundances in Bosten Lake. In Taitema Lake, *Triplophysa yarkandensis* (31.0%) and *C. auratus* (27.7%) were the two most abundant species.

**Figure 5:**
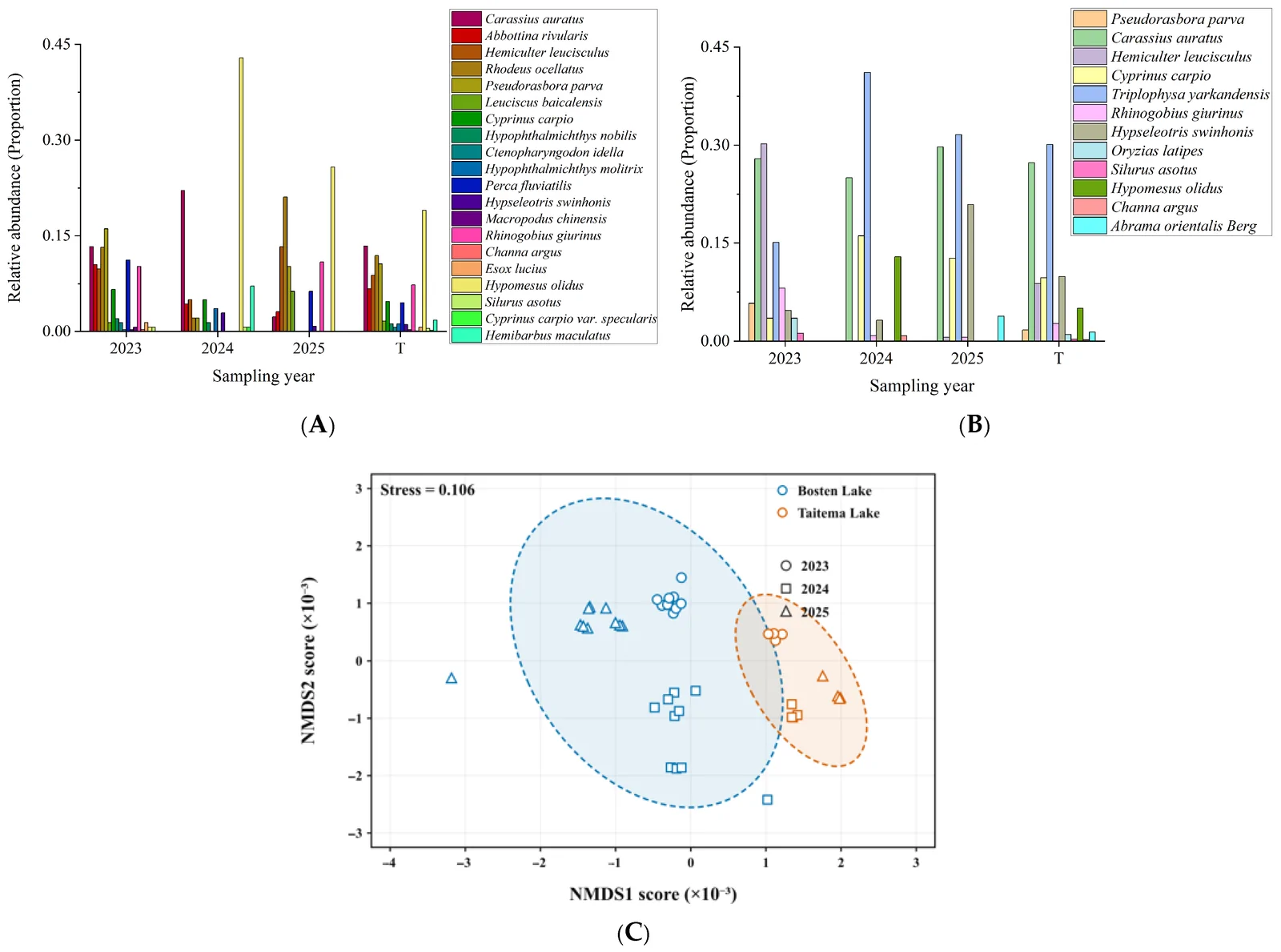
Relative abundance of fish in Bosten Lake (**A**) and Taitema Lake (**B**) from 2023 to 2025. “T” denotes the total cumulative relative abundance across the three study years. (**C**) Non-metric multidimensional scaling (NMDS) ordination of fish assemblages based on Bray–Curtis dissimilarities calculated from square-root-transformed relative species abundances. Each point represents one site × year assemblage (*n* = 42). Colors identify lakes and symbols identify sampling years. Dashed envelopes are descriptive 95% data ellipses calculated from the mean and covariance of the scores for each lake. The two-dimensional solution had stress = 0.106.

The annual community dynamics are shown in Figure 5. The community structure of Bosten Lake exhibited significant interannual fluctuations (Figure 5A): in 2023, Pseudorasbora parva had the highest relative abundance (16.1%), followed by *Carassius auratus, Rhodeus ocellatus, Perca fluviatilis, Rhinogobius giurinus, Abbottina rivularis, Hemiculter leucisculus,* and *Cyprinus carpio*, accounting for 13.3%, 13.2%, 11.2%, 10.2%, 10.5%, 9.8%, and 6.6% of the total fish abundance, respectively, while the relative abundances of other species were all below 5.0%. In 2024, the proportion of the non-native *Hypomesus olidus* increased abruptly to 42.9%, forming a dual-dominant assemblage together with *C. auratus* (22.1%), while *Hemibarbus maculatus, H. leucisculus,* and *C. carpio* accounted for 7.1%, 5.0%, and 5.0%, respectively, and the relative abundances of all other species were below 5.0%, indicating intensified community simplification. In 2025, the abundance of *H. olidus* decreased to 25.8%, while that of *R. ocellatus* increased to 21.1%; *H. leucisculus, R. giurinus, P. parva, Leuciscus baicalensis,* and *P. fluviatilis* accounted for 13.3%, 10.9%, 10.2%, 6.3%, and 6.3% of the total fish abundance, respectively, with all other species below 5.0%.

The interannual fluctuations in community structure were also pronounced in Taitema Lake (Figure 5B). In 2023, the non-native *Hemiculter leucisculus* had the highest relative abundance (30.2%), followed by *Carassius auratus, Triplophysa yarkandensis, Rhinogobius giurinus,* and *Pseudorasbora parva*, which accounted for 27.9%, 15.1%, 8.1%, and 5.8% of the total fish abundance, respectively, while the relative abundances of all other species were below 5.0%. In 2024, *Triplophysa yarkandensis* had the highest relative abundance (41.1%), followed by *C. auratus, Cyprinus carpio,* and *Hypomesus olidus*, accounting for 25.0%, 16.1%, and 12.9%, respectively, with all other species below 5.0%. In 2025, *T. yarkandensis* remained the most abundant species (31.6%), followed by *C. auratus, Hypseleotris swinhonis,* and *C. carpio,* which accounted for 29.7%, 20.9%, and 12.7%, respectively, while the relative abundances of all other species were below 5.0%.

The two-dimensional NMDS had stress = 0.106 (Figure 5C). In the reanalysed between-lake test, the three repeated observations from each site were retained as one whole-plot permutation unit. After adjustment for year, assemblage composition differed between lakes (pseudo-F1,38 = 37.92, partial R^2^ = 0.499, exact *p* = 0.0010; Table 1). Multivariate dispersion also differed between lakes (PERMDISP: F = 5.46, *p* = 0.030); therefore, the PERMANOVA result cannot be interpreted solely as centroid separation. With year labels permuted only within sites, year effects were detected in Bosten Lake (pseudo-F2,18 = 29.37, partial R^2^ = 0.765, *p* < 0.001) and Taitema Lake (pseudo-F2,6 = 156.36, partial R^2^ = 0.981, exact *p* = 0.0046). Temporal dispersion did not differ among years in either lake (Bosten *p* = 0.066; Taitema *p* = 0.282).

From 2023 to 2025, a total of eight dominant fish species (*D_i_* ≥ 0.10) were identified in Bosten Lake over the three-year period (Table 2). In 2023, the dominant species were *Abbottina rivularis* (0.10), *Carassius auratus* (0.13), *Perca fluviatilis* (0.11), *Pseudorasbora parva* (0.16), *Rhodeus ocellatus* (0.13), and *Rhinogobius giurinus* (0.10). In 2024, *C. auratus* and *Hypomesus olidus* were dominant, with dominance values of 0.22 and 0.43, respectively. In 2025, the dominant species were *Hemiculter leucisculus* (0.13), *H. olidus* (0.26), *P. parva* (0.10)*, R. ocellatus* (0.21), and *R. giurinus* (0.11).

From 2023 to 2025, a total of six dominant fish species (*D_i_* ≥ 0.10) were identified in Taitema Lake over the three-year period (Table 3). In 2023, the dominant species were *Carassius auratus* (0.28), *Hemiculter leucisculus* (0.30), and *Triplophysa yarkandensis* (0.15). In 2024, *T. yarkandensis* was the most dominant (0.41), followed by *C. auratus*, *Cyprinus carpio*, and *Hypomesus olidus*, with dominance values of 0.25, 0.16, and 0.13, respectively. In 2025, *T. yarkandensis* (0.32) continued to dominate, with dominance values of 0.30, 0.13, and 0.21 for *C. auratus*, *C. carpio*, and *Hypseleotris swinhonis*, respectively.

### 3.3. Species Diversity, Evenness, and Richness

**Figure 6:**
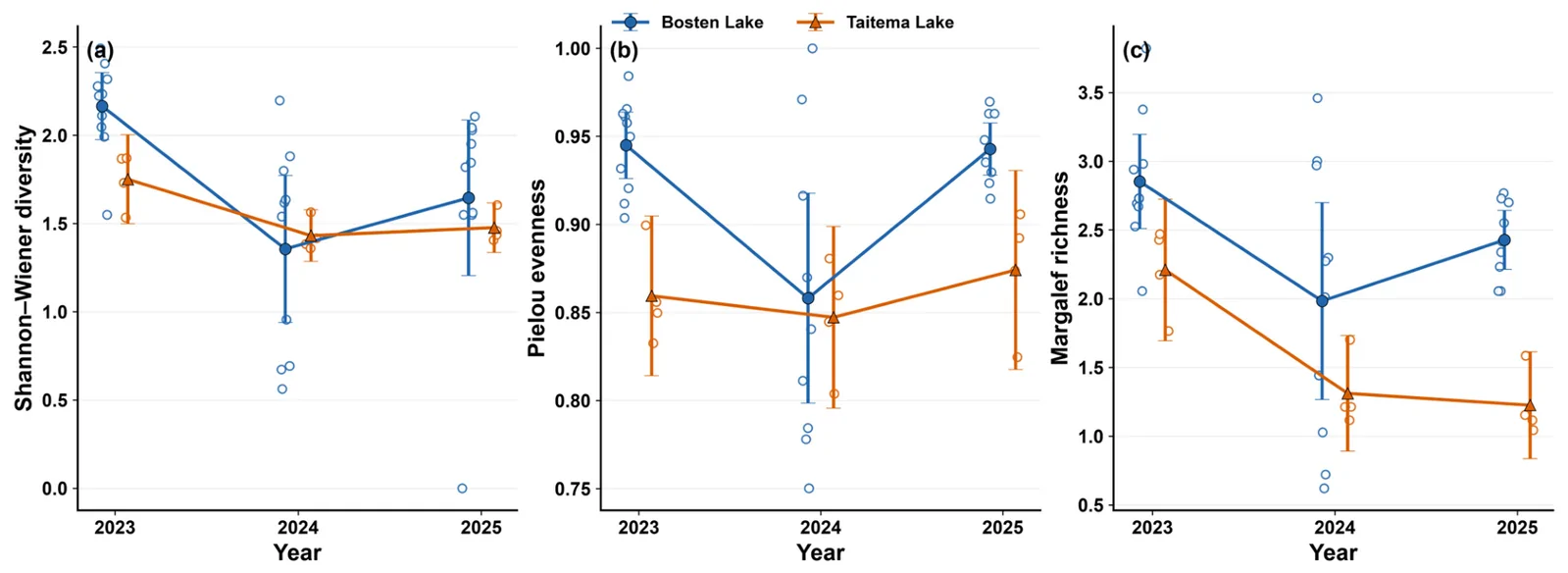
Temporal variation in fish diversity indices in Bosten Lake and Taitema Lake from 2023 to 2025. Open symbols represent site-level observations; filled symbols and error bars show observed annual means and 95% confidence intervals for (**a**) Shannon–Wiener diversity, (**b**) Pielou evenness, and (**c**) Margalef richness. Blue circles and orange triangles denote Bosten Lake and Taitema Lake, respectively. Mixed-effects models included lake and categorical year as fixed effects and site nested within lake as a random intercept. Lake effects (Taitema minus Bosten) were −0.169 (95% CI −0.673 to 0.335), −0.056 (−0.105 to −0.008), and −0.818 (−1.451 to −0.185), respectively.

Site-level Shannon–Wiener diversity and Pielou evenness decreased from 2023 to 2024 and increased in 2025 in both lakes. Margalef richness partially recovered in Bosten Lake but remained low and decreased slightly from 2024 to 2025 in Taitema Lake (Figure 6). After adjustment for year, Taitema Lake had lower Pielou evenness and Margalef richness, whereas the Shannon–Wiener contrast included zero (Table 4). The Shannon–Wiener model used 42 site-years; Pielou and Margalef used 41 because both indices were undefined for one single-species, single-individual site-year. Margalef is an index and should not be interpreted as raw species richness; year-specific raw-richness contrasts remained nonsignificant after Holm correction.

### 3.4. Fish Community Diversity and Its Correlation with Environmental Factors

**Figure 7:**
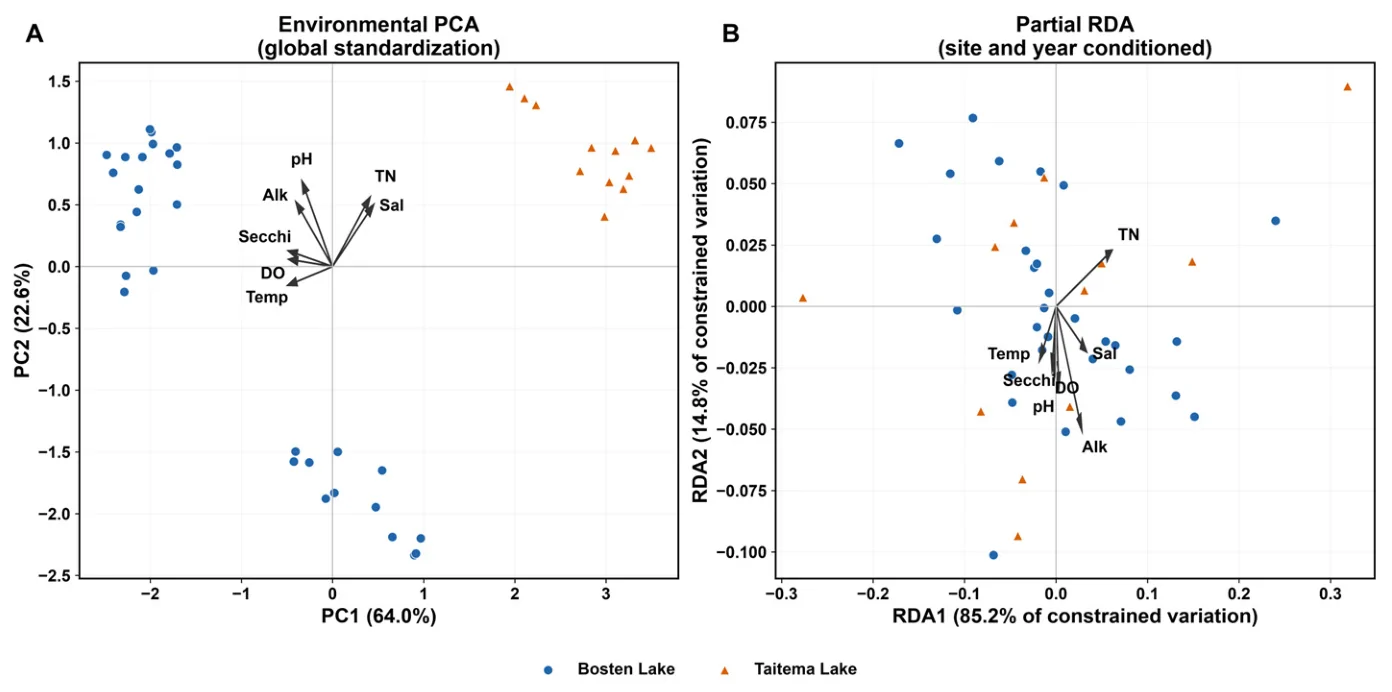
Descriptive environmental variation and the conditioned environmental–community analysis. (**A**) PCA of the seven retained environmental variables. Variables were not transformed and were globally centred and scaled across all 42 site-year observations; thus, between-lake mean differences were retained. (**B**) Partial RDA of Hellinger-transformed species abundances using PC1 and PC2 as predictors and conditioning on site and year. Conditioning on site removes persistent between-site variation, including the lake contrast; conditioning on year removes common annual effects. The test therefore concerns within-site temporal covariation (pseudo-F2,24 = 1.285, partial R^2^ = 0.097, *p* = 0.1754; 9999 permutations restricted within site). RDA-axis percentages are shares of constrained variation. Colours and symbols identify the source lake but are not a test of lake separation. Secchi, Secchi depth; Temp, water temperature; DO, dissolved oxygen; Sal, salinity; Alk, total alkalinity; TN, total nitrogen. Arrows are descriptive and do not represent independently significant effects.

The globally standardised environmental PCA retained between-lake mean differences (Figure 7A). PC1 and PC2 explained 64.0% and 22.6% of the total environmental variation, respectively. The separation of lake observations in this descriptive PCA reflects their overall physicochemical differences; it is not the hypothesis tested by the partial RDA.

After conditioning on site and year, only within-site temporal variation not shared as a common year effect remained for the partial RDA (Figure 7B); the persistent lake contrast was removed with site. RDA1 and RDA2 accounted for 85.2% and 14.8% of the constrained variation, respectively. The overall association between the two environmental PCA gradients and assemblage composition was nonsignificant (pseudo-F2,24 = 1.285, partial R^2^ = 0.097, restricted-permutation *p* = 0.1754). Accordingly, Figure 7B is interpreted only as an exploratory representation of within-site temporal environmental–community covariation and is not evidence that environmental differences explain the PERMANOVA lake effect.

Repeated-measures partial correlations were evaluated for the seven retained environmental variables and three diversity indices within each lake. Although some unadjusted coefficients were moderate to large, none remained significant after Benjamini–Hochberg correction within lake (all q > 0.05; Figure S1). The partial RDA test of within-site temporal environmental–community covariation was also nonsignificant (pseudo-F2,24 = 1.285, partial R^2^ = 0.097, *p* = 0.1754). Thus, the unadjusted coefficient directions are exploratory, and no retained environmental variable was statistically supported as an independent correlate of diversity or assemblage composition.

All models converged and were nonsingular. Shannon–Wiener residuals were non-normal (Shapiro–Wilk *p* < 0.001); Pielou and Margalef residuals were not (*p* = 0.515 and 0.258). Breusch–Pagan tests indicated heteroscedasticity for Pielou (*p* = 0.001) and Margalef (*p* = 0.016), but lake-specific residual-variance models gave nearly identical lake estimates (−0.056 and −0.815). Leave-one-site-out changes were <0.51 SE. Thus, the lake-effect conclusions were stable, although the Shannon–Wiener result requires caution (Figure S3).

## 4. Discussion

From 2023 to 2025, a total of 20 fish species belonging to 7 orders and 12 families were identified in Bosten Lake. Wang, et al. [19] recorded 17 fish species belonging to 4 orders and 8 families during spring, summer, and autumn surveys in 2019. Differences in survey duration and sampling effort limit direct inference of a temporal trend in species richness. Our cumulative three-year survey (2023–2025) recorded 20 species overall, representing a broadly similar total species pool to that reported historically. However, observed annual species richness within our study period decreased consistently from 18 species in 2023 to 13 in 2024 and 10 in 2025. In 2023, a total of 18 species were recorded, with Cyprinidae being the most species-rich family, among which *Pseudorasbora parva* had the highest relative abundance, accounting for 16.1% of the total fish abundance. In 2024, a total of 13 species were recorded, also dominated by Cyprinidae, but *Hypomesus olidus* became the most abundant species, reaching 42.9%. In 2025, a total of 10 species were recorded, still dominated by Cyprinidae, with *H. olidus* remaining the most abundant species (25.8%). From 2023 to 2025, a total of 12 fish species belonging to 6 orders and 9 families were identified in Taitema Lake. Our study revealed clear interannual variation in community composition, with measurable among-site variation within each year. In 2023, a total of 9 species were recorded, dominated by Cyprinidae, with *Hemiculter leucisculus* having the highest relative abundance (30.2%). In 2024, a total of 7 species were recorded, also dominated by Cyprinidae, but *Triplophysa yarkandensis* became the most abundant species (41.1%). In 2025, a total of 7 species were recorded, still dominated by Cyprinidae, with *T. yarkandensis* remaining the most abundant species (31.6%).

The multivariate analyses showed that the two lakes supported compositionally distinct fish assemblages, despite Cyprinidae being the most species-rich family in both. The whole-site permutation test showed a lake effect after adjustment for year (Bray–Curtis PERMANOVA: pseudo-F1,38 = 37.92, partial R^2^ = 0.499, exact *p* = 0.0010). Because lake-level dispersion also differed (PERMDISP: F = 5.46, *p* = 0.030), this result should not be interpreted solely as centroid separation. The differences in dominant fish species between the two lakes may be related to differences in hydrological regimes and introduction histories. Bosten Lake is the largest inland freshwater lake in China. Since the commencement of commercial fisheries in 1958 and the introduction of non-native fishes in 1962, the composition of fish resources in the lake has been in a state of continuous flux for more than half a century [19]. A survey in 2019 identified *Hemiculter leucisculus*, *Hypomesus olidus*, and *Carassius auratus* as dominant species in Bosten Lake [19]. The fish community in Bosten Lake has undergone four documented phases of compositional change over the past decades: a native fish phase, a phase dominated by *Carassius auratus* and *Cyprinus carpio*, a *Perca fluviatilis*-dominated phase, and the current *Hypomesus olidus*-dominated phase [13,19]. Continuous fish introductions have led to the successive replacement of non-native species in Bosten Lake, with *Hemiculter leucisculus* and *H. olidus* being recorded around 2000; a survey in 2019 showed that *H. leucisculus*, *H. olidus*, and *C. auratus* had become the absolute dominant species [19]. Studies have shown that although *H. leucisculus* and *H. olidus* are invasive species with similar ecological niches, they can coexist through spatial habitat segregation. *Hemiculter leucisculus* tends to inhabit shallow littoral zones when surface water temperatures rise in spring and summer, while *H. olidus* prefers pelagic waters, with the median habitat depth difference between the two species being approximately 1.7 m, and water temperature has been suggested as a factor associated with this spatial separation [20]. In contrast, Taitema Lake, as the terminal lake of the mainstream Tarim River and the Qarqan River, has its fish composition significantly influenced by ecological water conveyance projects. Taitema Lake dried up after the lower reaches of the Tarim River experienced flow cessation in 1972 [21,22], and its endemic fish fauna was subsequently lost in 1980 [23]. With the implementation of ecological water conveyance projects since 2000 [24,25], the water area of Taitema Lake has expanded considerably [25,26]. The native fish species in Taitema Lake are those brought in by concentrated water conveyance from the mainstream Tarim River and inflows from the Qarqan River, mainly including *Schizothorax biddulphi* and *Triplophysa yarkandensis* [27]; the lake is currently dominated by artificially stocked cultured non-native species (e.g., *C. auratus*, *Ctenopharyngodon idellus*, *Hypophthalmichthys molitrix*, and *Aristichthys nobilis*). Studies have suggested that H. leucisculus may have entered Taitema Lake from Bosten Lake through water conveyance projects for reproduction, and subsequently dispersed into the Tarim River [28]. The community in Taitema Lake is likely strongly influenced by external inputs, and its interannual variation is consistent with the characteristics of a recently restored system. Therefore, the observed patterns suggest a difference in compositional dynamics between these two study lakes: Bosten Lake is consistent with a history of long-term species introductions and interspecific competition, while Taitema Lake is consistent with rapid hydrological and environmental change associated with ecological water conveyance. Historical species introductions and hydrological changes provide plausible context for the observed patterns, but this observational study did not quantify stocking, dispersal, recruitment, or food-web processes, and therefore cannot assign the observed temporal variation to a single mechanism. A limitation of this study is that catches were not recorded separately by fishing gear, so gear-specific catch per unit effort (CPUE) cannot be calculated. However, an identical suite of fishing gears was deployed consistently at every sampling site across both lakes and all survey years, ensuring comparable relative sampling effort for cross-site and cross-lake comparisons. All cross-lake comparisons of species richness are supported by effort-standardized rarefaction and accumulation analyses; nevertheless, these rarefaction-based adjustments address sampling completeness but do not remove all potential gear-selectivity effects on relative abundance.

The environmental analyses did not identify a statistically supported independent correlate of fish diversity or assemblage composition. After accounting for site and year, none of the associations between the seven retained variables (Secchi depth, water temperature, pH, dissolved oxygen, salinity, total alkalinity, and total nitrogen) and the three diversity indices remained significant after Benjamini–Hochberg correction within either lake (all q > 0.05). The moderate-to-large unadjusted coefficients shown in Figure S1 are therefore exploratory and should not be interpreted as evidence of lake-specific environmental effects. Consistently, the partial RDA did not detect within-site temporal environmental–community covariation after site and year were conditioned out (pseudo-F2,24 = 1.285, partial R^2^ = 0.097, *p* = 0.1754). This model does not test whether environmental differences explain the between-lake PERMANOVA result. Likewise, none of the 77 coefficients between the 11 common species and the seven retained environmental variables remained significant after correction; these coefficients are not interpreted as evidence of physiological or ecological tolerance, and no species is designated as tolerant or sensitive. The two lakes differed descriptively in several measured environmental characteristics, but these contrasts do not establish that any retained variable independently drove the observed fish-community patterns. The three-year duration, the small number of Taitema Lake site-years, correlations among environmental variables, and unmeasured hydrological, habitat, stocking, and biotic factors limit mechanistic inference [15,29]. Longer verified environmental and hydrological time series are required to evaluate potential drivers [13].

## 5. Conclusions

From 2023 to 2025, 20 fish species were recorded in Bosten Lake and 12 in Taitema Lake, with Cyprinidae the most species-rich family in both systems. Bosten Lake had higher Margalef richness and Pielou evenness overall, whereas Shannon–Wiener diversity did not differ significantly between lakes; year-specific, site-level species richness contrasts were not significant after Holm correction. Bray–Curtis analyses showed strong compositional differentiation between lakes and significant temporal change within each lake, with beta-diversity partitioning confirming that much of the interannual change represented species replacement. Neither repeated-measures partial correlations nor partial RDA detected a robust association between the retained environmental variables and fish diversity or assemblage composition. The study therefore documents marked differences and temporal turnover in these two lakes but does not establish their environmental causes. These descriptive baseline data may inform fish resource monitoring and restoration planning in these two study systems, but further work is needed before generalizing to other arid-region lakes.

## Figures and Tables

**Table 1:** PERMANOVA formulas, permutation structures, and results.

Test	Formula	Permutation Structure	Pseudo-F	df	Partial R^2^	*p*
Lake, adjusted for year	Distance ~ Year + Lake	Exact whole-site permutations; all 1001 allocations preserving 10:4 sites; three years kept together	37.92	1, 38	0.499	0.0010
Year, Bosten Lake	Distance ~ Site + Year	Year permuted within site; 9999 restricted permutations	29.37	2, 18	0.765	<0.001
Year, Taitema Lake	Distance ~ Site + Year	Year permuted within site; all 1296 permutations	156.36	2, 6	0.981	0.0046

Note: Distance = Bray–Curtis dissimilarity calculated from square-root-transformed relative abundances. Lake was tested after year; year was tested after site.

**Table 2:** Dominant fish species in Bosten Lake from 2023 to 2025.

Order	Genus	Dominant Taxa	2023	2024	2025
Cypriniformes	Abbottina	*Abbottina rivularis*	0.10	-	-
Cypriniformes	Carassius	*Carassius auratus*	0.13	0.22	-
Cypriniformes	Hemiculter	*Hemiculter leucisculus*	-	-	0.13
Osmeriformes	Hypomesus	*Hypomesus olidus*	-	0.43	0.26
Perciformes	Perca	*Perca fluviatilis*	0.11	-	-
Cypriniformes	Pseudorasbora	*Pseudorasbora parva*	0.16	-	0.10
Cypriniformes	Rhodeus	*Rhodeus ocellatus*	0.13	-	0.21
Gobiiformes	Rhinogobius	*Rhinogobius giurinus*	0.10	-	0.11

Note: Dominance = annual catch of a species/total annual catch; Only species with dominance *D_i_* ≥ 0.10 are shown; “-“ denotes non-dominant or absent in that year.

**Table 3:** Dominant fish species in Taitema Lake from 2023 to 2025.

Order	Genus	Dominant Taxa	2023	2024	2025
Cypriniformes	Carassius	*Carassius auratus*	0.28	0.25	0.30
Cypriniformes	Hemiculter	*Hemiculter leucisculus*	0.30	-	-
Cypriniformes	Triplophysa	*Triplophysa yarkandensis*	0.15	0.41	0.32
Cypriniformes	Cyprinus	*Cyprinus carpio*	-	0.16	0.13
Osmeriformes	Hypomesus	*Hypomesus olidus*	-	0.13	-
Gobiiformes	Hypseleotris	*Hypseleotris swinhonis*	-	-	0.21

Note: Dominance was calculated as the annual number of individuals of a species divided by the total number of individuals captured in that year; Only species with dominance values *D_i_* ≥ 0.10 are listed; “-” indicates that the species was not a dominant species or did not occur in that year.

**Table 4:** Lake-effect estimates, 95% confidence intervals, variance components, and model diagnostics for the mixed-effects models.

Response	Lake Effect	95% CI	*p*	Site Variance	Residual Variance	Convergence/Singularity	Shapiro–Wilk *p*	Breusch–Pagan *p*
Shannon–Wiener	−0.169	−0.673 to 0.335	0.478	0.12636	0.07957	Converged; nonsingular	<0.001	0.506
Pielou	−0.056	−0.105 to −0.008	0.027	0.00087	0.00164	Converged; nonsingular	0.515	0.001
Margalef	−0.818	−1.451 to −0.185	0.016	0.17902	0.18458	Converged; nonsingular	0.258	0.016

Note: Lake effect = Taitema Lake minus Bosten Lake. Negative estimates indicate lower values in Taitema Lake. Shapiro–Wilk and Breusch–Pagan *p* values assess residual normality and homoscedasticity, respectively.

## Data Availability

The corresponding author can provide the data supporting the results of this study upon a reasonable request.

## Supplementary Materials

The following supporting information can be downloaded at: https://www.mdpi.com/article/10.3390/ani16193149/s1, Table S1: The total number of individuals collected per lake, year and sampling site; Table S2: Summary of retained environmental variables; Table S3: Species × site-year abundance matrix (number of individuals) for Bosten Lake and Taitema Lake, 2023–2025; Table S4: Site-year environmental dataset for Bosten Lake and Taitema Lake, 2023–2025. Figure S1. Repeated-measures partial correlations between retained environmental variables and fish-diversity indices in Bosten Lake (A) and Taitema Lake (B); Figure S2: Exploratory partial correlations between 11 common fish species and the seven retained environmental variables; Figure S3: Mixed-effects model diagnostics.
