# Supplementary material for: Fish Community Structure, Diversity, and Their Environmental Correlates in Two Arid-Region Lakes of Northwest China: Bosten Lake and Taitema Lake
Source: Animals (Basel). 2026 Oct 8;16(19):3149. doi: 10.3390/ani16193149 (PMC13663557; doi:10.3390/ani16193149)
Supplement: Supplementary file 1 [file animals-16-03149-s001.zip › animals-4550346-supplementary.pdf]

Table S1. The total number of individuals collected per lake, year and sampling site.

Lake	Year	Site													
		S1	S2	S3	S4	S5	S6	S7	S8	S9	S10	T1	T2	T3	T4
Bosten Lake	2023	41	20	47	13	30	56	16	27	39	7				
	2024	21	7	28	4	14	29	5	12	18	2				
	2025	18	7	23	6	13	28	7	13	12	1				
Taitema Lake	2023											25	17	27	17
	2024											36	27	34	27
	2025											46	36	44	32

Table S2. Summary of retained environmental variables.

Lake	Environmental variable	Unit	n	Mean ±SD	Minimum	Maximum	Range
Bosten Lake	Secchi depth	m	30	1.83 ± 0.68	1.01	2.99	1.98
	Water temperature	°C		26.09 ± 0.64	24.98	27.44	2.46
	pH	unitless		8.53 ± 0.41	7.76	9.00	1.24
	Dissolved oxygen	mg/L		7.56 ± 0.84	5.97	8.98	3.01
	Salinity	‰		0.37 ± 0.18	0.11	0.55	0.45
	Total alkalinity	mg/L as $CaCO_3$		191.17 ± 29.31	136.40	233.88	97.48
	Total nitrogen	mg/L		0.55 ± 0.16	0.33	0.92	0.59
Taitema Lake	Secchi depth	m	12	0.41 ± 0.11	0.34	0.64	0.30
	Water temperature	°C		24.12 ± 0.62	23.27	25.50	2.23
	pH	unitless		8.41 ± 0.09	8.25	8.56	0.31
	Dissolved oxygen	mg/L		5.94 ± 0.72	5.26	7.46	2.19
	Salinity	‰		5.22 ± 0.38	4.67	5.90	1.23
	Total alkalinity	mg/L as $CaCO_3$		169.33 ± 6.62	157.32	177.04	19.72
	Total nitrogen	mg/L		1.39 ± 0.19	1.14	1.68	0.53

Table note: Values summarize site-year observations. Bosten Lake comprised 30 observations from 10 stations over three years; Taitema Lake comprised 12 observations from four stations over three years. SD is the sample standard deviation. Range was calculated as maximum minus minimum.

Table S3. Species × site-year abundance matrix (number of individuals) for Bosten Lake and Taitema Lake, 2023–2025.

Bosten Lake	2023	S1	3	0	0	0	0	5	4	0	5	1	0	1	0	3	0	6	0	0	5	1	7	0
Bosten Lake	2023	S2	2	0	0	0	0	2	2	0	2	0	0	0	1	1	0	3	0	0	3	0	4	0
Bosten Lake	2023	S3	4	0	0	0	1	4	5	1	6	1	0	1	0	4	0	5	0	1	7	1	6	0
Bosten Lake	2023	S4	1	0	0	0	0	1	1	0	2	0	0	0	0	1	0	2	0	0	2	0	3	0
Bosten Lake	2023	S5	3	0	0	0	0	3	3	0	3	0	0	0	1	2	0	4	1	0	4	1	5	0
Bosten Lake	2023	S6	5	0	0	0	0	6	6	0	7	1	0	1	1	4	0	7	0	0	8	1	8	1
Bosten Lake	2023	S7	2	0	0	0	0	2	2	0	2	0	0	0	0	1	0	2	0	0	2	0	3	0
Bosten Lake	2023	S8	3	0	0	0	1	3	3	0	3	0	0	0	0	2	0	4	0	0	3	1	4	0
Bosten Lake	2023	S9	4	0	1	0	0	4	4	1	3	1	0	1	1	2	0	5	1	0	4	1	6	0
Bosten Lake	2023	S10	2	0	0	0	0	0	1	0	0	0	0	0	0	0	0	1	0	0	1	0	2	0
Bosten Lake	2024	S1	1	0	0	0	0	0	1	10	0	0	2	0	0	1	0	1	0	1	4	0	0	0
Bosten Lake	2024	S2	0	0	0	0	0	0	0	4	0	0	1	0	0	0	0	0	0	0	2	0	0	0
Bosten Lake	2024	S3	1	0	0	0	0	0	1	12	0	0	2	0	0	2	0	1	0	1	5	1	1	1
Bosten Lake	2024	S4	0	0	0	0	0	0	0	3	0	0	0	0	0	0	0	0	0	0	1	0	0	0
Bosten Lake	2024	S5	1	0	0	0	0	0	1	6	0	0	1	0	0	1	0	0	0	1	3	0	0	0
Bosten Lake	2024	S6	1	0	0	0	0	0	1	13	0	0	2	0	0	2	0	1	0	1	6	0	1	1
Bosten Lake	2024	S7	0	0	0	0	0	0	0	3	0	0	0	0	0	0	0	0	0	0	2	0	0	0
Bosten Lake	2024	S8	1	0	0	0	0	0	1	5	0	0	1	0	0	1	0	0	0	0	3	0	0	0
Bosten Lake	2024	S9	1	0	0	0	0	0	1	4	0	0	1	0	0	1	0	0	1	1	4	1	1	2
Bosten Lake	2024	S10	1	0	0	0	0	0	0	0	0	0	0	0	0	0	0	0	0	0	1	0	0	0

Bosten Lake	2025	S1	2	0	0	0	0	2	1	5	1	0	0	0	1	0	0	3	0	0	1	0	2	0
Bosten Lake	2025	S2	1	0	0	0	0	1	0	2	0	0	0	0	0	0	0	2	0	0	0	0	1	0
Bosten Lake	2025	S3	3	0	0	0	0	2	1	6	2	0	0	0	2	0	0	4	0	0	1	0	2	0
Bosten Lake	2025	S4	1	0	0	0	0	1	0	2	0	0	0	0	0	0	0	1	0	0	0	0	1	0
Bosten Lake	2025	S5	2	0	0	0	0	1	1	3	1	0	0	0	1	0	0	3	0	0	0			
Bosten Lake	2025	S6	3	0	0	0	0	3	1	7	2	0	0	0	2	0	0	5	0	0	1			
Bosten Lake	2025	S7	1	0	0	0	0	1	0	2	0	0	0	0	0	0	0	2	0	0	0			
Bosten Lake	2025	S8	2	0	0	0	0	2	0	3	1	0	0	0	1	0	0	3	0	0	0			
Bosten Lake	2025	S9	2	0	0	0	0	1	0	3	1	0	0	0	1	0	0	3	0	0	0			
Bosten Lake	2025	S10	0	0	0	0	0	0	0	0	0	0	0	0	0	0	0	1	0	0	0			
Taitema Lake	2023	T1	8	0	0	4	0	2	0	0	0	0	0	0	0	1	1	0	0	0	7			
Taitema Lake	2023	T2	6	0	0	3	0	1	0	0	0	0	0	0	0	0	0	0	0	0	5			
Taitema Lake	2023	T3	7	0	0	4	0	2	0	0	0	0	0	0	0	1	1	0	1	0	8			
Taitema Lake	2023	T4	5	0	0	2	0	2	0	0	0	0	0	0	0	1	1	0	0	0	4			
Taitema Lake	2024	T1	0	0	0	15	0	0	0	5	0	0	0	0	0	6	0	0	0	0	9			
Taitema Lake	2024	T2	0	0	0	12	0	0	0	3	0	0	0	0	0	4	0	0	0	0	7			
Taitema Lake	2024	T3	0	0	1	13	0	1	0	4	0	0	0	0	0	5	0	0	0	0	9			
Taitema Lake	2024	T4	0	0	0	11	0	0	0	4	0	0	0	0	0	5	0	0	0	0	6			
Taitema Lake	2025	T1	0	2	0	14	0	0	0	0	0	0	0	0	0	6	0	0	0	0	14			
Taitema Lake	2025	T2	0	1	0	12	0	0	0	0	0	0	0	0	0	4	0	0	0	0	11			
Taitema Lake	2025	T3	1	2	0	13	0	1	0	0	0	0	0	0	0	5	0	0	0	0	13			
Taitema Lake	2025	T4	0	1	0	11	0	0	0	0	0	0	0	0	0	5	0	0	0	0	9			

Notes. Each row represents one site-year sampling unit (Bosten Lake: 10 sites × 3 years = 30 rows; Taitema Lake: 4 sites × 3 years = 12 rows; total = 42 site-year observations). Cell values are the number of individuals of each species captured; 0 indicates absence. Native/introduced status is assigned at the Tarim Basin scale. Triplophysa yarkandensis is the only native species recorded in this survey.

Table S4. Site-year environmental dataset for Bosten Lake and Taitema Lake, 2023–2025.

Lake	Year	Site	Sechi_depth_(m)	Water_temperature (℃)	pH	Dissolved_oxygen_(mg/L)	Salinity_(‰)	Total_alkalinity (mg/L_as_$CaCO_3$)	Total_nitrogen_(mg/L)
Bosten	2023	S1	2.46	25.51	8.99	8.00	0.51	214.48	0.62
Bosten	2023	S10	1.18	25.56	8.16	7.55	0.12	171.65	0.37
Bosten	2023	S2	1.27	25.03	7.97	6.24	0.11	146.76	0.47
Bosten	2023	S3	1.15	25.29	8.31	7.70	0.25	150.85	0.47
Bosten	2023	S4	1.32	24.98	7.99	6.09	0.10	179.82	0.44
Bosten	2023	S5	2.47	25.59	8.80	7.64	0.48	216.41	0.68
Bosten	2023	S6	2.97	25.98	8.72	7.84	0.50	214.16	0.62
Bosten	2023	S7	1.41	26.40	8.89	8.78	0.48	211.50	0.44
Bosten	2023	S8	2.60	26.04	8.85	7.54	0.53	196.08	0.64
Bosten	2023	S9	1.81	26.12	8.75	8.70	0.46	222.71	0.33
Bosten	2024	S1	2.31	25.67	8.94	7.92	0.53	208.87	0.67
Bosten	2024	S10	1.19	25.92	8.19	7.50	0.12	173.90	0.40

Bosten	2024	S2	1.22	25.45	7.82	6.05	0.12	156.71	0.48
Bosten	2024	S3	1.01	25.81	8.33	7.80	0.27	137.06	0.43
Bosten	2024	S4	1.27	25.83	7.93	6.19	0.11	168.34	0.52
Bosten	2024	S5	2.58	25.93	8.72	7.59	0.53	233.88	0.61
Bosten	2024	S6	2.99	26.49	8.89	7.47	0.49	222.50	0.57
Bosten	2024	S7	1.44	26.59	8.81	8.98	0.48	215.28	0.40
Bosten	2024	S8	2.27	26.45	8.82	7.55	0.54	214.43	0.90
Bosten	2024	S9	1.82	26.94	8.63	8.43	0.48	212.11	0.38
Bosten	2025	S1	2.35	26.09	8.96	7.75	0.55	218.85	0.71
Bosten	2025	S10	1.15	26.14	8.27	7.63	0.13	162.15	0.42
Bosten	2025	S2	1.18	25.50	7.85	6.12	0.12	153.49	0.51
Bosten	2025	S3	1.01	26.22	8.30	7.81	0.28	136.40	0.47
Bosten	2025	S4	1.30	25.95	7.76	5.97	0.11	169.57	0.56
Bosten	2025	S5	2.68	26.25	8.88	7.81	0.53	209.38	0.83
Bosten	2025	S6	2.79	26.99	8.85	7.34	0.53	219.46	0.62

Bosten	2025	S7	1.23	27.44	8.92	8.27	0.51	188.03	0.46
Bosten	2025	S8	2.58	27.29	8.85	7.97	0.54	208.21	0.91
Bosten	2025	S9	1.65	27.20	8.72	8.46	0.51	201.99	0.32
Taitema	2023	T1	0.34	23.55	8.48	5.70	5.30	177.03	1.14
Taitema	2023	T2	0.62	24.36	8.56	6.53	5.43	175.28	1.34
Taitema	2023	T3	0.35	23.27	8.34	5.47	4.67	170.72	1.23
Taitema	2023	T4	0.37	23.62	8.46	5.38	5.14	171.10	1.28
Taitema	2024	T1	0.33	23.82	8.34	5.72	5.01	162.21	1.14
Taitema	2024	T2	0.63	24.75	8.51	7.45	5.74	166.36	1.55
Taitema	2024	T3	0.38	23.89	8.30	5.81	4.68	168.32	1.42
Taitema	2024	T4	0.37	23.60	8.38	5.72	4.93	159.68	1.40
Taitema	2025	T1	0.34	24.14	8.36	5.50	5.42	157.31	1.65
Taitema	2025	T2	0.51	25.50	8.50	7.19	5.90	174.34	1.60
Taitema	2025	T3	0.35	24.44	8.41	5.51	5.05	174.41	1.26
Taitema	2025	T4	0.33	24.46	8.25	5.26	5.29	175.16	1.67

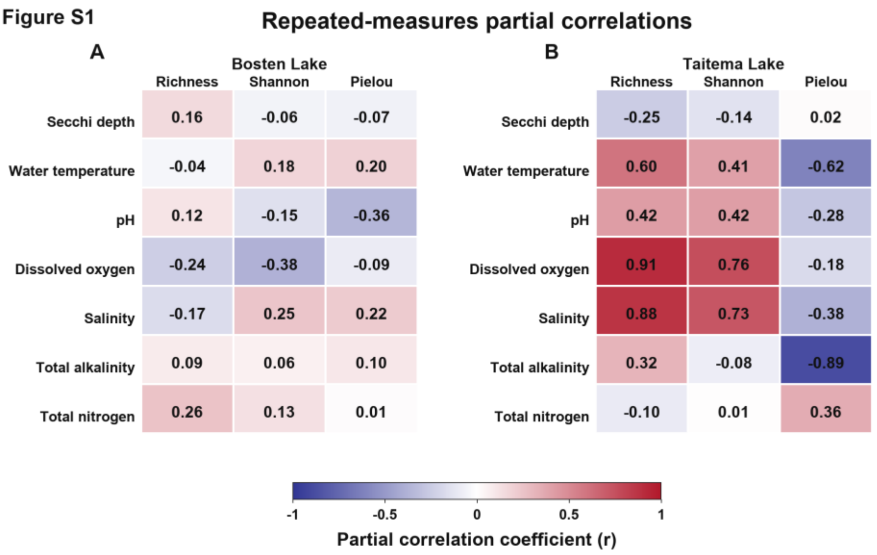

Figure S1. Repeated-measures partial correlations between retained environmental variables and fish-diversity indices in Bosten Lake (A) and Taitema Lake (B). Values are Pearson correlation coefficients after controlling for station and year. P values were obtained from 9,999 permutations restricted within station and adjusted within each lake across the 21 tests using the Benjamini–Hochberg false-discovery-rate procedure. No correlation met $q < 0.05$.

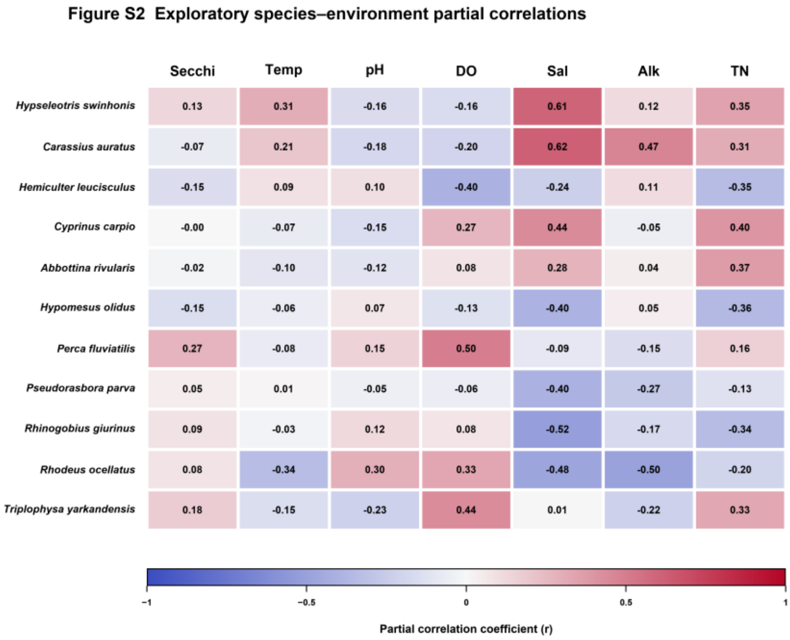

Figure S2. Exploratory partial correlations between 11 common fish species and the seven retained environmental variables. Values are partial Pearson correlation coefficients calculated from Hellinger-transformed site-year species relative abundances after controlling for site and categorical year across all 42 site-year observations. Secchi, Secchi depth; Temp, water

temperature; pH, pH; DO, dissolved oxygen; Sal, salinity; Alk, total alkalinity; TN, total nitrogen. P values were obtained from 9,999 permutations restricted within site and adjusted across all 77 species–variable tests using the Benjamini–Hochberg procedure. No relationship remained significant after correction (all q > 0.05). These coefficients were not used to classify taxa as tolerant or sensitive. Only species occurring in at least eight site-year samples and represented by at least 20 individuals were included.

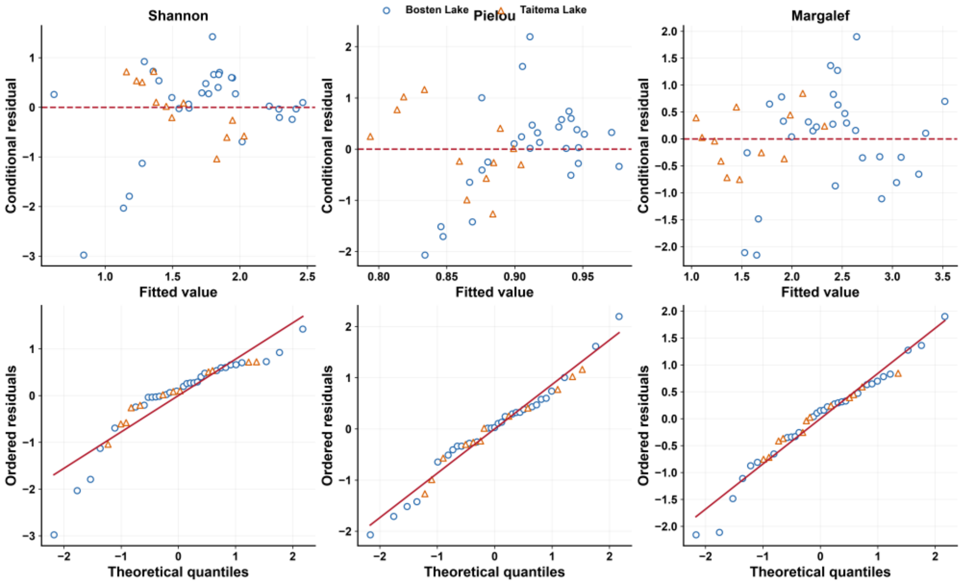

Figure S3. Mixed-effects model diagnostics. The upper panels show standardized conditional residuals against fitted values; the lower panels show normal Q–Q plots for the Shannon–Wiener, Pielou, and Margalef models. Blue circles and orange triangles denote Bosten Lake and Taitema Lake, respectively.
